# A True Human Tail In A Neonate Born In Saudi Arabia: A Case Report

**DOI:** 10.7759/cureus.53346

**Published:** 2024-01-31

**Authors:** Sarah A Algallaf, Majed A Alobaid, Shahad G Alghamdi, Kholoud S Alanazi

**Affiliations:** 1 Pediatrics, Jubail Health Network, Jubail, SAU; 2 Neonatology, Jubail Health Network, Jubail, SAU; 3 Orthopedics, College of Medicine, Imam Abdulrahman Bin Faisal University, Dammam, SAU; 4 Pediatrics, Dammam Maternity and Children Hospital, Dammam, SAU

**Keywords:** kingdom of saudi arabia (ksa), case report pediatric, spinal dysraphism, magnetic resonance imaging, neonate, tail

## Abstract

A caudal cutaneous appendage known as the true human tail is a rare and benign condition. Different classification systems have been established, mostly based on the presence of associated spinal dysraphism. Imaging studies play an important role in detecting the prognosis and developing a management plan. Here, we present a rare case of a true human tail with no underlying spinal dysraphism in a preterm neonate.

## Introduction

The human tail is a rare congenital anomaly that manifests as a protruding midline lesion of the lumbosacrococcygeal region [1]. It is considered a vestigial remnant of the embryonic tail that normally regresses by the eighth week of gestation [2]. The etiology of the human tail is not fully understood, but it may be related to genetic or environmental factors [1]. Human tails are classified into two types: true tails and pseudo tails [2]. True tails consist of skin, connective tissue, adipose tissue, muscles, blood vessels, nerves, and mechanoreceptors, and they can exhibit spontaneous or reflex movements. Pseudo tails are secondary protrusions caused by various anomalies or neoplasms, such as spinal dysraphism, lipoma, teratoma, chondrodystrophy, or a parasitic fetus. The presence of a human tail may be associated with other congenital malformations, especially of the spinal cord and column. Therefore, a thorough neurological examination and neuroimaging studies, such as MRI, are essential to exclude any underlying abnormalities and plan the appropriate surgical management. In this case report, we present a rare case of a true human tail with spinal bifida in a newborn male. We describe the clinical features, radiological findings, histopathological examination, and surgical outcome of this unique case.

## Case presentation

The patient is a newborn male infant delivered via cesarean section at 33 weeks of gestational age with a low birth weight of 2.4 kg. He was delivered at our hospital with an Apgar score of 7/10 at one and five minutes, respectively. The patient was admitted to the NICU due to mild respiratory distress in the form of tachypnea, nasal flaring, and intercostal and subcostal retractions; there was no grunting or cyanosis. Apart from this, he was admitted to exclude spinal dysraphism and the evaluation and management of the cutaneous congenital anomaly arising from the back in the lumbosacral region. He had no other congenital defects. He also had an unremarkable antenatal history, and there was no family history of any developmental or congenital anomalies.

**Figure 1:**
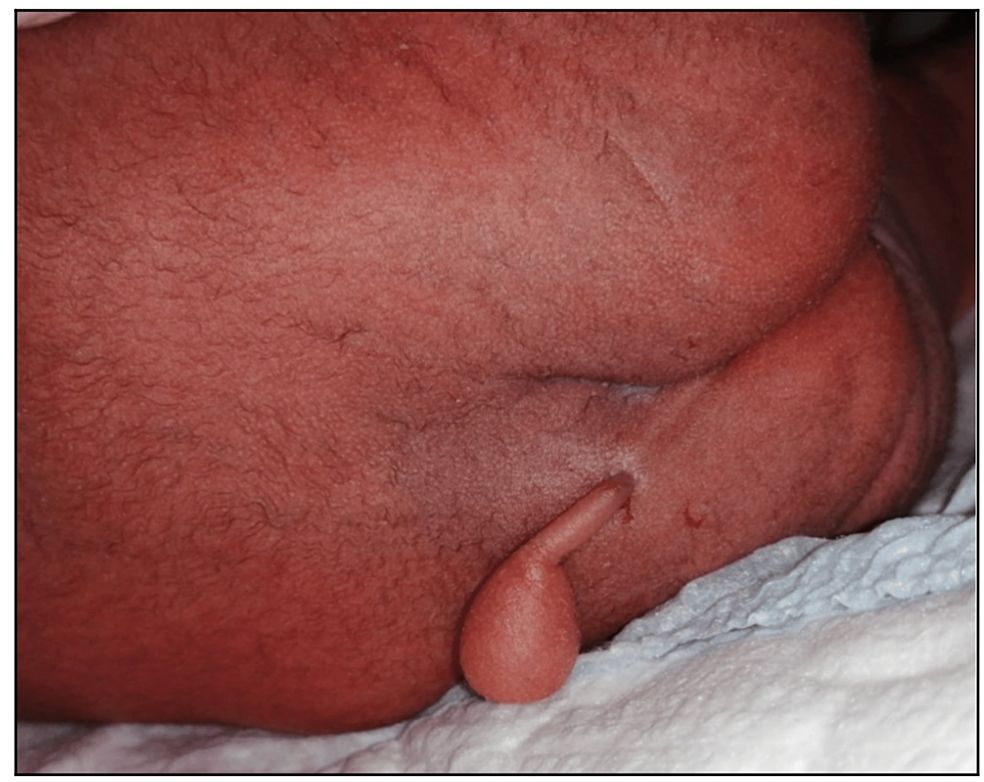
Clinical photograph of a true human tail arising from the patient's sacrococcygeal region

On examination, he was active pink in color and vitally stable, with no dysmorphic features, normal limbs, and normal external male genital. The lumbosacral examination found a 3 cm-long cutaneous soft tissue appendage covered by normal skin arising and hanging down from the mid-sacral region (Figure 1). The mass was soft, firm, non-tender, and not translucent at the left gluteal cleft with a circular-shaped end.

A detailed physical examination revealed no other associated anomalies. The cutaneous soft tissue appendage was a 3 cm-long, cylindrical, pink, soft, and mobile mass arising from the mid-sacral region. It was non-tender and did not bleed on palpation. There was no spontaneous movement of the appendage. There was no evidence of spinal dysraphism, tethered cord syndrome, or spina bifida. The patient's lab investigations (CBC, renal function test (RFT), liver function test (LFT)) were within the normal range. He was managed with nasal oxygen on the first day and was weaned off gradually; other than that, the patient was managed with routine NICU care.

**Figure 2:**
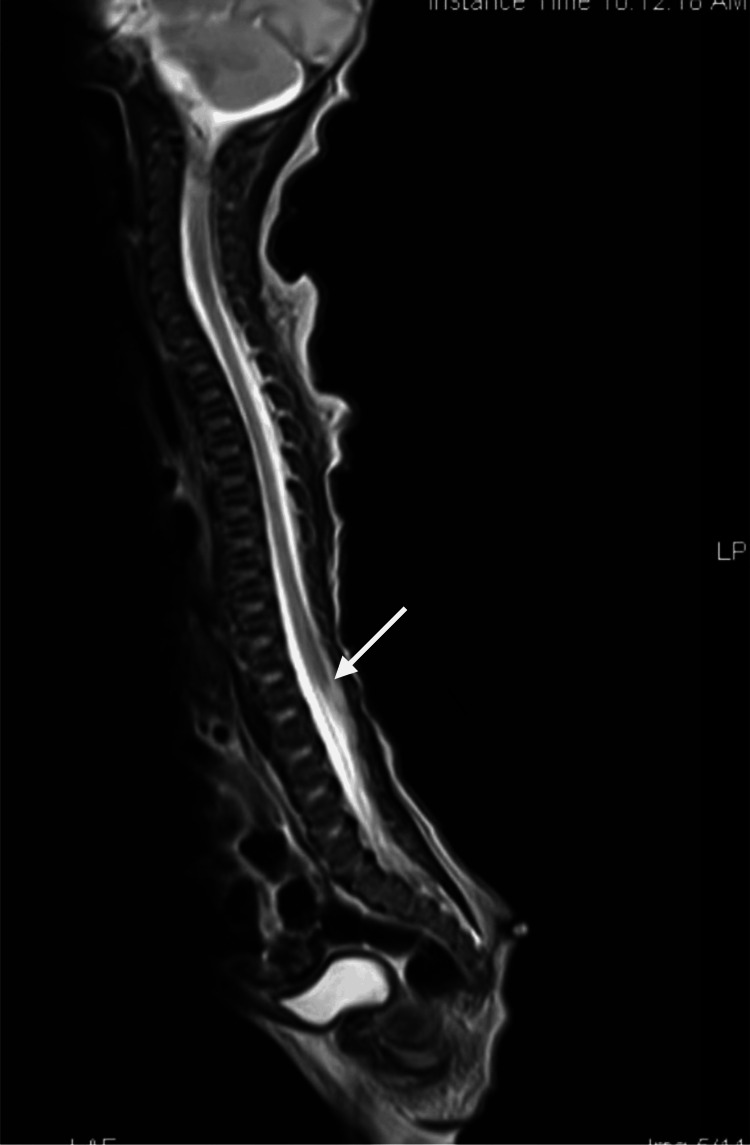
MRI of the spine shows the normal end level of the conus medullaris (arrow)

**Figure 3:**
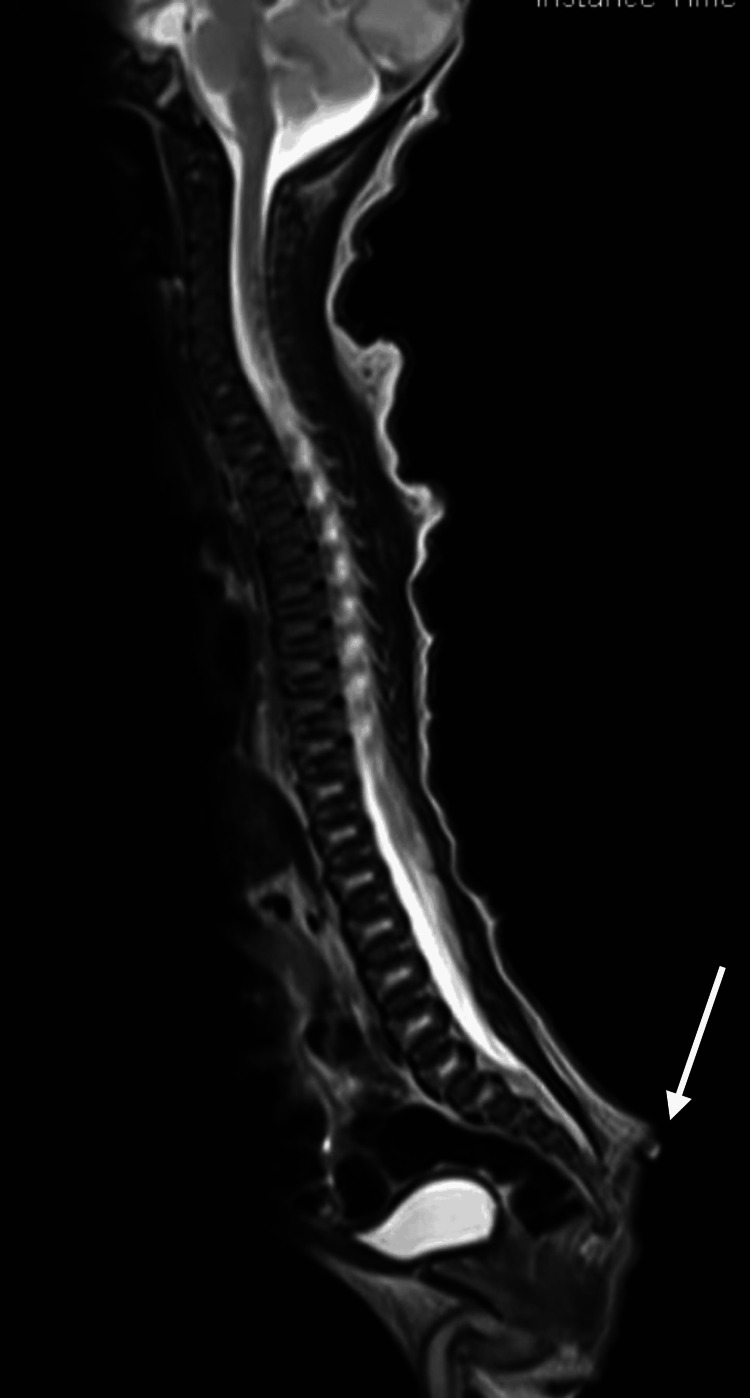
MRI of the spine shows a pedunculated appendage with fat intensity hanging from the mid-sacral region (arrow)

An MRI of the spine revealed an approximately 3 cm (about 1.18 in) long pedunculated appendage with fat intensity arising and hanging down from the mid-sacral region. There was no definite communication with the thecal sac, no obvious spinal dysraphism, and the conus was located at the level of the first lumbar vertebra, which was suggestive of a true human tail. In addition, the cord and theca displayed a normal course and signal pattern, and the scanned vertebrae displayed a normal signal as well as a cortical outline (Figures 2-3).

The patient also had an MRI of the brain, which showed multiple bilateral foci in the periventricular white matter that elicited high signal intensity in flair and showed restricted diffusion. These findings are suggestive of periventricular leukomalacia. Other findings were unremarkable and within the normal range for his age.

**Figure 4:**
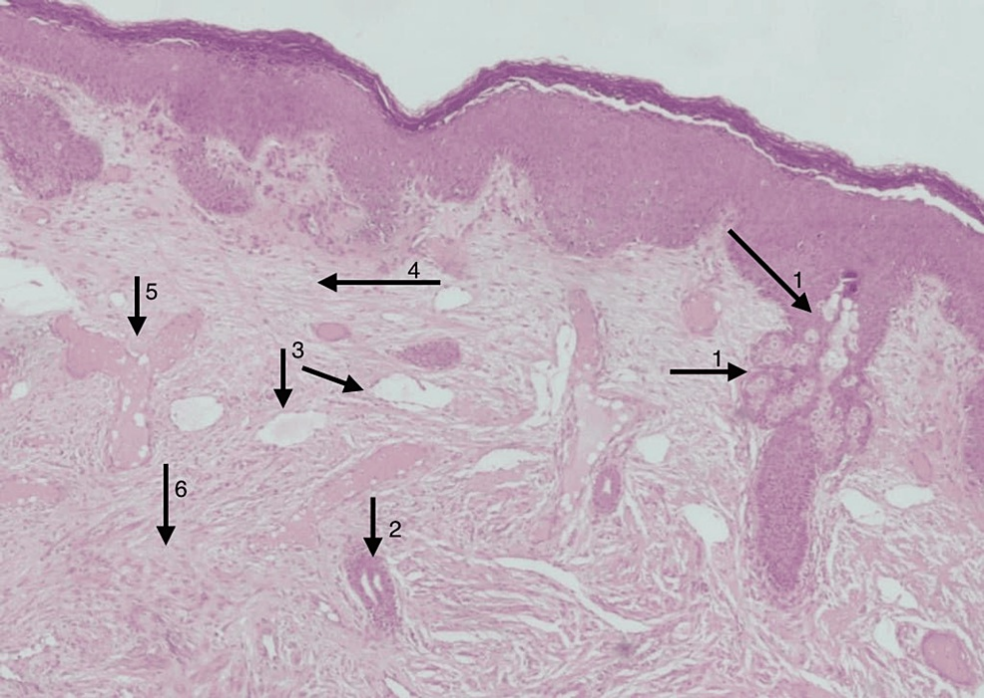
Microscopic examination of the skin and tissue of the true tail 1: Adnexal structures including hair follicles, 2: Sweat and sebaceous glands, 3: Dense connective tissue with congested blood vessels, 4: Islands of adipose tissue, 5: Nerve bundles, 6: Bands of smooth muscle fibers

The patient underwent excision of the tail; microscopic examination showed skin with adnexal structures including hair follicles, sweat, and sebaceous glands. The underlying tissue revealed dense connective tissue with congested blood vessels, islands of adipose tissue, nerve bundles, and some bands of smooth muscle fibers. No bones or cartilaginous tissue were found. No malignant cells were detected (Figure 4). The diagnosis was made from the pathohistological specimen which concluded that it is a skin tag consistent with a true vestigial tail.

## Discussion

The caudal appendage, i.e., the 'human tail', is a rare benign congenital anomaly that results in a vestigial lumbosacral dorsal cutaneous appendage classified as true tails or pseudo tails [3]. A clinical, radiological, and histopathological examination can differentiate between them, as well as the presence of underlying spinal dysraphism [4]. Several embryological techniques have been proposed regarding the etiology of the human tail. According to the most common theory, the human tail results from incomplete regression of the most distal end of the normal embryonic tail in the developing fetus, indicating that the human tail is an abnormality in embryonic development [5,6]. 

A true tail is a distal boneless midline protrusion composed of adipose, connective tissue, vessels, nerves, and striated muscle covered by normal skin [4,7]. Whereas pseudo tails are varied lesions that contain teratoma, lipoma, and myelomeningocele, having in common a lumbosacral protrusion and a superficial resemblance to persistent vestigial tails [7]. In our case, it corresponds with a true tail, as it was not associated with other congenital malformations. Also, it arose in the most frequent region, the sacrococcygeal region.

More recently, Tojima and Yamada proposed a new precise classification of the human tail in which they classified it into two main entities based on its content and location (Table 1) [8]. The type 1 human tail contains bone and cartilage; further, they are subdivided based on the coccyx characteristic (Ia is a human tail caused by the protrusion of the coccyx, and Ib is a human tail with bony or cartilaginous non-coccygeal elements). Type 2 human tail, as in our case, doesn’t contain bones or cartilage and is also subdivided per its location, i.e., positioned higher or lower the natal cleft [8]. In our case, the neonate had type IIa, which is the most common type.

**Table 1:** Classification of human tails or caudal appendages

Classifications	Subdivision	
Type I contains bones or cartilage	Type Ia: Bones or cartilage form part of the coccyx	
	Type Ib: Bones or cartilage do not form part of the coccyx	
Type II does not contain bones or cartilage	Type IIa: Tail situated higher than natal cleft	
	Type IIb: Tail situated lower than natal cleft	

The human tail is frequently encountered in males and is not inherited, but familial cases have been described in the literature where three generations of the same family were born with true tails [9,10]. However, in our case, there was no family history of human tails, and the male infant had a normal antenatal period. In some cases, true tails sometimes have spontaneous or reflexive movement, while voluntary contractions are unusual [7,8]. In our case, the tail was immobile. Mostly, the human tail arises from the midline, but in our case, the tail was located on the left side [8]. Other anomalies most frequently associated with this type of human tail are dystrophic spinal cord malformations, including lipoma, myelomeningocele, and less frequently meningocele [8,11]. 

Preoperative diagnostic imaging, such as MRI, plays a crucial role in identifying associated anomalies, predicting the prognosis, and establishing appropriate operative management. The prognosis of our case was excellent once spinal dysraphism was ruled out by MRI and was managed with a simple resection and regular follow-up.

## Conclusions

In the medical literature, only a few cases of human tails have been reported. The current case describes the true human tail of a neonate, as evidenced by the absence of underlined spinal dysraphism. Imaging studies are crucial for the assessment and diagnosis of a true tail. This is a benign condition that can be surgically treated by simple excision.
